# Data-driven prioritization and preclinical evaluation of therapeutic targets in glioblastoma

**DOI:** 10.1093/noajnl/vdaa151

**Published:** 2020-11-05

**Authors:** Cyrillo G Brahm, U Kulsoom Abdul, Megan Houweling, Myra E van Linde, Tonny Lagerweij, Henk M W Verheul, Bart A Westerman, Annemiek M E Walenkamp, Rudolf S N Fehrmann

**Affiliations:** 1 Department of Medical Oncology, University of Groningen, University Medical Center Groningen, Groningen, The Netherlands; 2 Department of Medical Oncology, Cancer Center Amsterdam, Brain Tumor Center Amsterdam, Amsterdam University Medical Centers, Location VUmc, Amsterdam, The Netherlands; 3 Department of Neurosurgery, Cancer Center Amsterdam, Brain Tumor Center Amsterdam, Amsterdam University Medical Centers, Location VUmc, Amsterdam, The Netherlands; 4 Department of Medical Oncology, Radboud University Medical Center, Nijmegen, The Netherlands

**Keywords:** glioblastoma, MAPK9, RRM2, therapeutic targets, XIAP

## Abstract

**Background:**

Patients with glioblastoma (GBM) have a dismal prognosis, and there is an unmet need for new therapeutic options. This study aims to identify new therapeutic targets in GBM.

**Methods:**

mRNA expression data of patient-derived GBM (*n* = 1279) and normal brain tissue (*n* = 46) samples were collected from Gene Expression Omnibus and The Cancer Genome Atlas. Functional genomic mRNA profiling was applied to capture the downstream effects of genomic alterations on gene expression levels. Next, a class comparison between GBM and normal brain tissue was performed. Significantly upregulated genes in GBM were further prioritized based on (1) known interactions with antineoplastic drugs, (2) current drug development status in humans, and (3) association with biologic pathways known to be involved in GBM. Antineoplastic agents against prioritized targets were validated in vitro and in vivo.

**Results:**

We identified 712 significantly upregulated genes in GBM compared to normal brain tissue, of which 27 have a known interaction with antineoplastic agents. Seventeen of the 27 genes, including *EGFR* and *VEGFA*, have been clinically evaluated in GBM with limited efficacy. For the remaining 10 genes, *RRM2*, *MAPK9* (*JNK2*, *SAPK1a*), and *XIAP* play a role in GBM development. We demonstrated for the MAPK9 inhibitor RGB-286638 a viability loss in multiple GBM cell culture models. Although no overall survival benefit was observed in vivo, there were indications that RGB-286638 may delay tumor growth.

**Conclusions:**

The MAPK9 inhibitor RGB-286638 showed promising in vitro results. Furthermore, in vivo target engagement studies and combination therapies with this compound warrant further exploration.

**Key Points**

- There is an unmet need for novel therapeutic targets in glioblastoma (GBM).
- We identified RRM2, MAPK9, and XIAP as novel potential therapeutic targets in GBM.
- The MAPK9 inhibitor RGB-286638 shows promising in vitro results.

**Importance of the Study**

Despite optimal first-line treatment of newly diagnosed glioblastoma (GBM), recurrence is inevitable and universally results in death in most cases. Therefore, there is a need for more effective treatment strategies and new therapeutic targets. Here, we applied functional genomic mRNA (FGmRNA) profiling, a method that corrects gene expression profiles for physiological and experimental factors irrelevant to the observed tumor phenotype, on publicly available microarray expression data of patient-derived GBM samples and normal brain tissue samples. Based on the class comparison of FGmRNA profiles, known interactions with antineoplastic drugs and the association with pathways involved in GBM carcinogenesis, we identified RRM2, MAPK9, and XIAP as potential therapeutic targets in GBM. Out of the available drugs targeting RRM2, MAPK9, and XIAP, the MAPK9 inhibitor RGB-286638 showed promising in vitro results warranting further exploration.

Glioblastoma (GBM) is the most common and aggressive primary brain tumor in adults. Currently, the standard first-line treatment for patients with newly diagnosed GBM consists of maximal surgical resection followed by postoperative radiation with concomitant and adjuvant temozolomide therapy. A randomized phase 3 trial in GBM patients, who had completed standard chemoradiotherapy, reported that adding tumor treating fields to maintenance temozolomide chemotherapy prolonged progression-free survival (7.1 months) and overall survival (OS; 20.5 months) as compared to controls (4.0 and 15.6 months, respectively).^1^ Unfortunately, despite optimal first-line treatment, recurrence is still almost inevitable. The prognosis of these patients remains poor with a median survival of 12–20 months.^2,3^

At the time of recurrence, treatment options are limited due to limitations in the use of surgery and re-irradiation, as well as the limited efficacy of systemic treatment.^1,4–6^ In the past decades, research focused on the molecular genetic profiles of GBM to provide insights into the pathogenesis of GBM and the tenacious resistance to conventional and targeted therapies. The Cancer Genome Atlas Research Network (TCGA) made a significant contribution and performed a comprehensive genomic and transcriptomic analysis on 206 GBM samples. They demonstrated relevant genomic alterations in the p53, retinoblastoma (Rb), and receptor tyrosine kinase (RTK)/phosphoinositide 3-kinase (PI3k) signaling pathways.^7^ Furthermore, unsupervised hierarchical clustering analysis of the TCGA GBM expression data linked transcriptomic alterations on the mRNA level with distinct molecular subtypes of GBM, which were confirmed on the single-cell transcriptomic level.^8–10^ Collectively, these data helped to identify frequently amplified genes in GBM, including *EGFR*, *PDGFRA*, *MET*, *CDK4*, and *PIK3CA*, and commonly deleted genes, such as *PTEN* and *RB1*.^7,11,12^ Genomic alterations can translate into downstream effects, such as changes in protein structures (with gain or loss of function) or changes of gene expression levels (with activation or inactivation of a gene or pathway).^13^ Therefore, genomic alterations (eg, somatic copy number alterations [SCNAs]) hold valuable information on the biological behavior of GBM, its resistance mechanisms to conventional therapy, and possible new therapeutic targets.

The method of functional genomic mRNA (FGmRNA) profiling demonstrated that the expression level of all genes can be affected by SCNAs. However, this effect is often subtle and obscured mainly by major, non-genetic factors (eg, physiological, metabolic, and experimental factors). FGmRNA profiling is capable of correcting gene expression data for these factors, resulting in a residual gene expression signal that highly correlates with SCNAs.^14^ Thus, FGmRNA profiling is capable of capturing the downstream effects of genomic alterations on gene expression levels.

We hypothesize that FGmRNA profiling of publicly available, raw microarray expression data of patient-derived GBM samples and normal brain tissue harbors valuable new insights on the downstream effects of genomic alterations in GBM. Therefore, this proof of concept study used FGmRNA profiling, followed by prioritization, to identify highly expressed genes in GBM with known drug interactions that could serve as new potential therapeutic targets. Subsequently, we investigated the preclinical antitumor activity of targeted agents directed against these potential therapeutic targets in GBM.

## Materials and Methods

Detailed methods information is provided in Supplementary Methods.

### Data Acquisition

We collected publicly available raw microarray expression data from the Gene Expression Omnibus (GEO). We obtained gene expression data from GEO for samples that were processed on the HG-U133A (GPL96) and HG-U133 plus 2.0 (GPL570) Affymetrix platforms. Simple Omnibus Format Text (SOFT) files were downloaded for both platforms. These SOFT files contain information on the samples as provided by the investigator who uploaded the data to GEO. To identify GBM samples, we first applied automated filtering with GBM-related keywords on the SOFT files (Supplementary Table S1). This search strategy was aimed at sensitivity to minimize the chance of missing relevant samples. Therefore, manual curation was necessary to remove all non-relevant and false-positive samples. Cell lines, cultured samples, and postmortem or animal tissues were excluded. In addition, we collected raw gene expression data from the TCGA GBM multiforme data set. These data were generated with the Affymetrix HT HG-U133A and were integrated with the GEO data set. Preprocessing and aggregation of raw data were performed according to the robust multi-array average algorithm. Quality control of the resulting expression data was executed, as previously described.^15^

### Class Comparison

FGmRNA profiling was used to capture the downstream effect of genomic alterations at gene expression levels. For a detailed description of FGmRNA profiling, we refer to the work of Fehrmann et al.^14^ We used the FGmRNA profiles of healthy brain tissue and GBM tissue to perform a genome-wide class comparison analysis. A Welch’s *T*-test was used to identify genes with differential FGmRNA expression. To assess the degree of multiple testing, we performed our analysis within a multivariate permutation test (1.000 permutations) with a false discovery rate of 1% and a confidence level of 99%. This resulted in a list of significantly associated genes, which contains no more than 1% false positives.

### Prioritization of Upregulated Genes

We manually curated the list of significantly upregulated genes to exclude duplicate results of multiple probes targeting the same gene, nonspecific probes mapping to multiple genes and probes that did not map to a known gene. Subsequently, we explored the resulting list of genes with the use of the Drug–Gene Interaction Database (DGIDb; http://www.dgidb.org/). The DGIDb integrates data of disease-relevant human genes, drugs, and proven or potential drug–gene interactions from 13 primary sources.^16^ This allowed us to select upregulated genes that interact with antineoplastic agents. We assessed all antineoplastic agents per gene with an additional PubMed and www.clinicaltrials.gov search to determine the mechanism of drug–gene interaction and the current drug development status in humans. Genes interacting with antineoplastic agents currently tested in various malignancies were prioritized. Furthermore, we assessed these prioritized genes with www.genecards.org and PubMed to identify their biological pathways. Ultimately, we selected the genes with a biological pathway involved with GBM carcinogenesis and a known interaction with antineoplastic agents tested in clinical cancer trials.

### In Vitro Experiments: Cell Lines and Description of Proliferation Assay

The established GBM cell lines U87, U251, T98G, and U138 were acquired from the ATCC and were cultured in Dulbecco’s modified Eagle’s medium supplemented with 5% FBS. The HT29 colorectal cell line was included as a control. The GBM8 primary cell culture was kindly provided by Dr. Bakhos Tannous (Harvard/MGH). Glioblastoma sphere cultures (GSCs) were obtained from single patient surgical specimens at MD Anderson and the Amsterdam University Medical Centers, location VUmc.^17^ GBM8 and GSCs were cultured at 37°C in Neurobasal-A Medium (Life Technologies) and supplemented with N2 (Life Technologies), B27 without vitamin A (Life Technologies), Glutamax (Life Technologies), human EGF (Tebu Bio), human FGF basic (Tebu Bio), heparin (Leo Pharma), and penicillin/streptomycin (Sigma).^18^ Cells were certified mycoplasma free by regular testing http://www.microbiome.nl/. RGB-286638 (Bio-Connect) was dissolved in DMSO to prepare a 20 mM stock solution.

For the U87, U251, T98G, and U138 GBM cell lines, response to RGB-286638 was assessed by 3-(4,5-dimethylthiazol-2-yl)-2,5-diphenyl-2H-tetrazolium bromide (MTT) assay as described previously.^19^ In short, cells were seeded on a transparent flat-bottom 96-well plate in a density of 2000 cells per well and were allowed to adhere for 24 h. After 24 h of incubation, at = 0, the measurement was carried out, and cells were treated with 100 µL of drug solution according to a concentration dilution series ranging from 0 to 20 µM. Subsequently, after 72 h of treatment, 100 µL of MTT indicator dye (5 mg/mL) was added to the wells, and cells were incubated for 2 h at 37°C. After the addition of 100 µL of 10% SDS/0.01 M HCl to the wells, absorption was measured at 540 nm in a microplate reader. The reading from the wells with cells cultured in control medium-plus DMSO was used as a 100% viability value. For GBM8 primary and GSC cell cultures, response to RGB-286638 was determined through CellTiter-Glo 3D assay to measure the ATP content of viable cells. Cells were plated at an optimal density (which varied between GSC lines) in 384-well plates 24 h prior to drug treatment. Subsequently, cells were exposed to a serial dilution of RGB-286638 for 72 h in triplicate. Cell viability was determined using CellTiter-Glo 3D (Promega). Relative light units (RLUs) were measured using the Tecan’s Connect microplate stacker, and RLUs were normalized against the DMSO controls.^20^ All experiments were performed in triplicate and repeated at least 2 times. Levels of response (ie, complete response, incomplete response, or resistance) were based on the viability at the highest concentration of RGB-286638 and the IC_50_. Thresholds were below or higher than 5% viability for complete versus incomplete responses and below or higher than an IC_50_ of 1 μM for incomplete responses versus resistance.

### Orthotopic In Vivo Mouse Model

Female athymic nude-Fox1nu mice (age 6–8 weeks; Envigo) were maintained in accordance with animal welfare guidelines and regulations of the VU University in Amsterdam, The Netherlands. GBM8 cells, stably expressing Firefly luciferase and mCherry, were intracranially injected in a volume of 5 µL (0.5 × 10^6^ cells) into the striatum, as previously described.^18^ Tumor engraftment and growth were determined by measuring the Firefly luciferase (Fluc) activity with a CCD camera after an intraperitoneal injection of 150 µL of d-luciferin (Gold Biotechnology). Fluc activity was measured 5 days after intracranial injection of GBM8 cells and subsequently 1–2 times a week during treatment and follow-up for a maximum total amount of 15 times. Mice with incomplete tumor engraftment (Fluc activity <10^4^ RLU) were excluded from the experiment. Based on the Fluc activity, mice were subsequently stratified into 3 treatment groups of 7 animals each and were all treated for 5 consecutive days with (1) Vehicle (PBS: 200 µL/day), (2) the pan-CDK inhibitor Flavopiridol (5 mg/kg/day) as a control for CDK inhibition, or (3) RGB-286638 (40 mg/kg/day). The treatment dose for RGB-286638 (40 mg/kg/day) was based on the in vivo study of Cirstea et al.^21^ in a multiple myeloma mice model. RGB-286638 and Flavopiridol were administered intravenously by tail vein. Experiments were performed under ethical review permission (AVD114002017841) and are reported according to the ARRIVE guidelines.

## Results

### Sample Identification

Following automated filtering, manual curation, removal of duplicates, and quality control, mRNA expression data of 46 normal brain tissue samples and 1279 patient-derived GBM samples were included for further analysis. In-depth information on the GEO and TCGA samples and their corresponding citations are provided in Supplementary Table S2.

### Class Comparison Between Normal Brain Tissue and Clinical GBM Samples

We identified 712 significantly upregulated and unique genes using a class comparison analysis between FGmRNA profiles of normal brain tissue samples and GBM samples (false discovery rate 1%, confidence level 99%). Detailed results are provided in Supplementary Table S3.

### Prioritization of Druggable Genes

**Figure 1.**
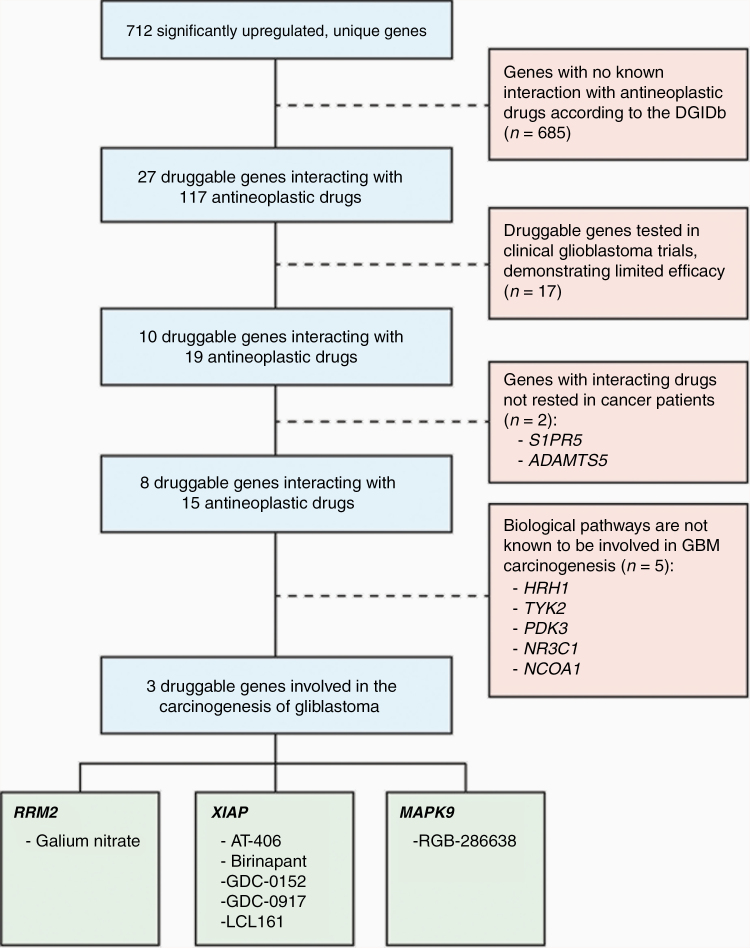
Prioritization process of druggable genes.

Of the 712 upregulated genes, 27 genes interacted with 116 antineoplastic drugs, according to the DGIDb (Table 1). Seventeen of the 27 druggable genes, including *EGFR* and *VEGFA*, were previously tested in clinical GBM trials and demonstrated limited efficacy (Supplementary Table S4). Therefore, these genes were excluded from further review. For the 10 remaining genes, 14 of the 20 interacting antineoplastic drugs are currently being tested in clinical trials for various cancers but have not been tested in clinical trials for GBM (Table 2). Drugs interacting with *S1PR5* and *ADAMTS5* are currently not being evaluated in patients with cancer according to www.clinicaltrials.gov and were therefore also excluded. For the remaining 8 genes (*HRH1*, *TYK2*, *RRM2*, *MAPK9*, *PDK3*, *XIAP*, *NR3C1*, and *NCOA1*), an additional literature search in Pubmed and http://www.genecards.org/ was performed, which identified *RRM2*, *MAPK9*, and *XIAP* as members of biological pathways that play an important role in the development of GBM (Figure 1).

**Table 1.** Significantly Upregulated Genes Interacting With Antineoplastic Drugs

Rank	Gene Symbol	Gene Title	Drug
2	*EGFR*	Epidermal growth factor receptor	Afatinib | Crizotinib | Dacomitinib | Erlotinib | Gefitinib | Icotinib | Lapatinib | Mubritinib | Neratinib | Pelitinib | Poziotinib | Vandetanib | Brigatinib | Canertinib | Rociletinib | Carboplatin | Cisplatinum | Paclitaxel | Sirolimus | Cetuximab | Panitumumab | Geldanamycin | AEE788 | AZD8931 | BIBX 1382 | BMS-599626 | BPIQ-I | CUDC-101 | PD 158780 | PD 174265
36	*HRH1*	Histamine receptor H1	Tesmilifene | Tranilast
59	*VEGFA*	Vascular endothelial growth factor A	Apatinib | Axitinib | Bevacizumab | Bevasiranib | Brivanib | Cabozantinib | Cediranib | Dovitinib | Endostatin-(84–114)-NH2 | Golvatinib | Linifanib | Lenvatinib | Lenalidomide | Motesanib | Nintedanib | Pazopanib | Pegaptanib | Ponatinib | Ranibizumab | Regorafenib | Semaxanib | Sorafenib | Sunitinib | Thalidomide | Tivozanib | Telatinib | Vandetanib | Vatalanib | Ziv-Aflibercept | 4SC-202 | ABT-510 | AEE788 | AZD2932 | BMS-794833 | CYC116 | ENMD-2076 | KI8751 | KRN633 | LY2874455 | MGCD-265 | RAF265 | SKLB1002 | SU5402 | TAK-593 | ZM306416 | ZM323881
77	*GPER*	G protein-coupled estrogen receptor 1	Fulvestrant | Tamoxifen
147	*PSMA2*	Proteasome subunit, alpha 2	Bortezomib | Carfilzomib
163	*TUBA3C*	Tubulin, alpha 3c	CYT997 | Epothilone B
232	*TYK2*	Tyrosine kinase 2	AT9283
319	*RRM2*	Ribonucleotide reductase M2	Gallium nitrate
369	*MAPK9*	Mitogen-activated protein kinase 9	RGB-286638
381	*MAPK1*	Mitogen-activated protein kinase 1	Erlotinib | Purvalanol A
399	*PDK3*	Pyruvate dehydrogenase kinase, isozyme 3	CPI-613
417	*PSMC2*	Proteasome 26S subunit, ATPase 2	Bortezomib | Carfilzomib | MLN9708
432	*DDR1*	Discoidin domain receptor tyrosine kinase 1	Imatinib
434	*SRD5A1*	Steroid-5-alpha-reductase, alpha polypeptide 1	Finasteride
440	*WEE1*	WEE1 G2 checkpoint kinase	MK-1775
450	*IL8*	Interleukin 8	ABT-510
458	*PSMD2*	Proteasome 26S subunit, non-ATPase 2	Bortezomib | Carfilzomib | Oprozomib
470	*S1PR5*	Sphingosine-1-phosphate receptor 5	Fingolimod
498	*ADAMTS5*	ADAM metallopeptidase with thrombospondin type 1 motif 5	Batimastat
507	*CFLAR*	CASP8 and FADD-like apoptosis regulator	Bicalutamide | Cabozantinib | Dovitinib | Nintedanib
513	*CYP3A5*	Cytochrome P450 family 3 subfamily A member 5	Erlotinib | Lovastatin
540	*MMP14*	Matrix metallopeptidase 14	Marimastat
577	*XIAP*	X-linked inhibitor of apoptosis	AT-406 | AZD5582 | Birinapant | GDC-0917 | GDC-0152 | LCL161 | SM-337
639	*NOTCH1*	Notch 1	MK-0752 | RO4929097
661	*NR3C1*	Nuclear receptor subfamily 3, group C, member 1 (glucocorticoid receptor)	Fluoxymesterone | Megestrol | Onapristone
664	*CDK6*	Cyclin-dependent kinase 6	Flavopiridol | LY2835219 | Palbociclib | RGB-286638 | Ribociclib
708	*NCOA1*	Nuclear receptor coactivator 1	Genistein

**Table 2.** Significantly Upregulated Genes and Interacting Antineoplastic Drugs Tested in Clinical Trials for Various Cancers

Rank	Gene	Interacting Drug	Interaction	Status	Tumor Type(s)
36	*HRH1*	Tesmilifene	Antagonist	Phase 2	Breast cancer
		Tranilast	Inhibitor	Phase 2	Prostate cancer
232	*TYK2*	AT9283	Inhibitor	Phase 2	Advanced solid tumors, ALL, AML, childhood solid neoplasms, CML, MDS, multiple myeloma, non-Hodgkin lymphoma
319	*RRM2*	Gallium nitrate	Inhibitor	Phase 2	Advanced solid tumors, cervical cancer, colorectal cancer, non-Hodgkin lymphoma, NSCLC, prostate cancer, SCLC, transitional cell carcinoma
369	*MAPK9*	RGB-286638	Inhibitor	Phase 1	Advanced solid tumors
399	*PDK3*	CPI-613	N/A	Phase 2	AML, colorectal cancer, MDS, multiple myeloma, non-Hodgkin lymphoma, SCLC
470	*S1PR5*	Fingolimod	N/A	None	None
498	*ADAMTS5*	Batimastat	N/A	None	None
577	*XIAP*	AT-406	Antagonist	Phase 1	Advanced solid tumors, AML
		AZD5582	Antagonist	None	None
		Birinapant	Antagonist	Phase 2	Advanced solid tumors, ALL, AML, MDS, ovarian cancer
		GDC-0917	Antagonist	Phase 1	Advanced solid tumors
		GDC-0152	Inhibitor	Phase 1	Advanced solid tumors, non-Hodgkin lymphoma
		LCL161	Inhibitor	Phase 2	Advanced solid tumors, breast cancer
		SM-337	N/A	None	None
661	*NR3C1*	Fluoxymesterone	Antagonist	Phase 2	Breast cancer
		Megesterol	Antagonist	Phase 3	Breast cancer
		Onapristone	Antagonist	Phase 2	Breast cancer, prostate cancer
708	*NCOA1*	Genistein	N/A	Phase 3	Prostate cancer

### The Potential Therapeutic Targets *RRM2*, *MAPK9*, and *XIAP*

Ribonucleotide reductase regulatory subunit M2 (*RRM2*) contributes to the upregulation of ribonucleotide reductase activity during the S phase of the cell cycle. It plays an essential role in regulating the total rate of DNA synthesis.^22,23^ The gene is implicated in temozolomide therapy resistance and is transcriptionally co-activated by *BRCA1*, protecting cells from endogenous replication stress, DNA damage, and apoptosis.^24–26^ Subsequently, in vitro and in vivo studies with inhibition of *RRM2* expression showed a significant decrease in tumor growth in various tumors, including GBM, and improved animal survival.^27^

The protein encoded by mitogen-activated protein kinase 9 (*MAPK9*), also known as c-Jun N-terminal kinase 2 (*JNK2*), is involved in regulating various cellular processes, including cell growth, transformation, and apoptosis.^28^ This pathway can also be activated by growth factors, such as epidermal growth factor and platelet-derived growth factor.^29–31^ Targeted inhibition of *MAPK9* with specific antisense oligonucleotides resulted in marked growth suppression in human GBM T98 cells, suggesting that *MAPK9* inhibition could have therapeutic benefit.^32^

The X-linked inhibitor of apoptosis protein (XIAP) is a strong caspase-binding protein and inhibits both the intrinsic and extrinsic apoptosis pathways.^33^ Overexpression of XIAP has been linked to tumor recurrence in prostate cancer and resistance to systemic and targeted therapy in breast cancer cells.^34–36^ Interestingly, the second mitochondria-derived activator of caspases (Smac) mimetics, which neutralizes XIAP, can sensitize GBM cells for temozolomide and can prime them for apoptosis.^37,38^

Based on the known drug–gene interactions and the current status of clinical evaluation in patients, the prioritized agents of interest are gallium nitrate as an inhibitor of RRM2, RGB-286638, as an inhibitor of MAPK9, and AT-406, Birinapant, GDC-0152, GDC-0917, and LCL161 as inhibitors of XIAP. Subsequently, the preclinical efficacy of these drugs was evaluated in GBM.

### The Sensitivity of GBM Cell Lines to Antineoplastic Agents Targeting MAPK9, RRM2, and XIAP

**Figure 2.**
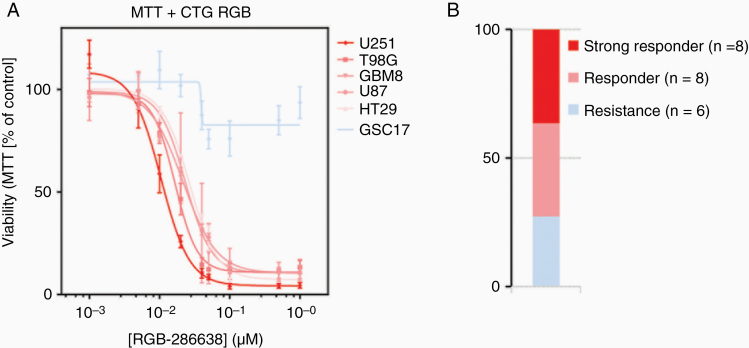
(A) Viability assay of exposure to MAPK9 inhibitor RGB-286638 for 72 h and (B) the total number of GBM cell cultures responding or resistant to RGB-286638.

All cell lines, including the colorectal cell line HT29 as a control and the GBM8 primary cell culture, were exposed to various concentrations of the drugs of interest (ranging from 0 to 20 µM) for 72 h. Exceptionally, for gallium nitrate, higher concentrations up to 2 mM were necessary. Interestingly, low exposure to RGB-286638 demonstrated near-complete inhibition in all cell lines (IC_50_ ranging from 0.01 to 0.03 µM; Figure 2), compared to all the other drugs in which high concentrations resulted in no or limited inhibition (Supplementary Figure S1A–F). Additional analyses in GBM sphere culture models, which resemble GBM heterogeneity more accurate, showed that about one-third of the tumor models showed a similarly high sensitivity to RGB-286638, and around one-third showed an incomplete response resulting in 5–30% viability after exposure to 1 µM (Supplementary Figure S2). Therefore, based on its antitumor activity, RGB-286638 was selected for preclinical evaluation in an orthotopic in vivo model.

### The Efficacy of RGB-286638 in an Orthotopic GBM8 Primary GBM In Vivo Model

**Figure 3.**
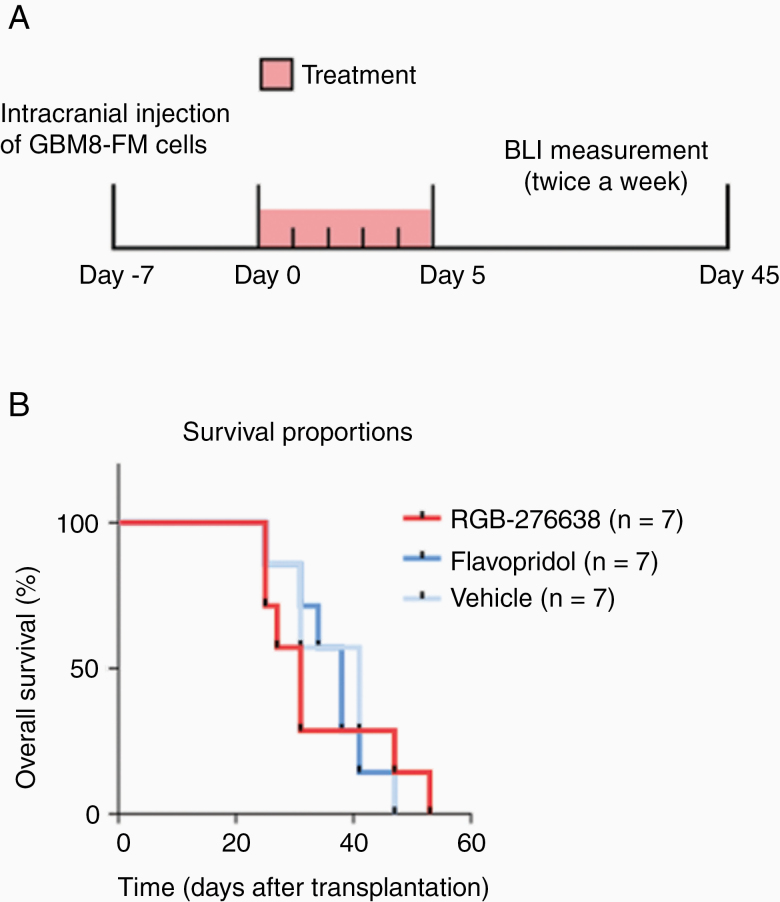
(A) Schematic overview of the in vivo experiment and (B) median overall survival in days of each treatment group.

The antitumor efficacy of RGB-286638 in an orthotopic in vivo mouse model, using primary GBM8 cells (Figure 3A), was studied. As RGB-286638 is an inhibitor of MAPK9, but also inhibits multiple cyclin-dependent kinases (CDKs), the pan-CDK inhibitor Flavopiridol was used as an extra control group. Orthotopic growth was assessed with in vivo luminescence measurement, which showed a significantly lower signal in mice treated with RGB-286638 compared to the vehicle and Flavopiridol group (*P* = 5.5 × 10^–8^ and *P* = .01, respectively). Flavopiridol did not show significantly lower signals compared to the vehicle-treated animals. In comparison to the vehicle control, no significant difference in median OS was observed for the animals treated with RGB-286638 (47 vs 53 days, respectively, *P* = .93; Figure 3B). These combined results indicate that RGB-286638 may delay tumor growth, possibly by its MAPK9 and CDK inhibitory properties. Overall, all treatments were well tolerated based on the animals’ conditions and weight during the follow-up period. However, some of the animals treated with RGB-286638 developed local skin lesions on their tail as a possible reaction to the administration via tail vein injection. The skin lesions in these mice recovered successfully after the topical administration of an antibacterial ointment. Therefore, the endpoint of this animal study was not limited by toxicity.

## Discussion

We applied FGmRNA profiling to correct gene expression data for the effect of non-genetic and experimental factors on gene expression levels, followed by data-driven prioritization to identify *RRM2*, *MAPK9*, and *XIAP* as new potential therapeutic targets in GBM. The preclinical efficacy of several clinical available compounds targeting RRM2, MAPK9, and XIAP was studied in GBM.

FGmRNA profiling is a method that corrects gene expression data for major, non-genetic factors (eg, physiological, metabolic, cell-type-specific, and experimental factors). The FGmRNA profiles enabled us to capture the downstream effect of SCNAs at gene expression levels. With this innovative method, we discovered *MAPK9* as a potential therapeutic target. Subsequently, we used RGB-286638, a small molecule to target MAPK9. As with most small-molecule kinase inhibitors, this compound has multiple targets, including the family of CDKs.^21,39^ Interestingly, low concentrations of RGB-286638 (ie, 100 nM) showed near-complete inhibition of viability in all classical GBM cell lines and approximately one-third of all primary GSC cultures. RGB-286638 has been clinically evaluated in a phase I study in patients with advanced solid tumors.^40^ In this phase I study, RGB-286638 was well tolerated in a dose of 120 mg/day for 5 consecutive days every 28 days. Furthermore, prolonged disease stabilization, ranging from 2 to 14 months, was seen across the different dose levels. Unfortunately, although RGB-286638 showed a consistent decrease in tumor growth, as determined by the luciferase signal in our orthotropic GBM8 mouse model, it did not result in a better OS.

We also investigated gallium nitrate, a simple gallium salt used for the treatment of cancer-related hypercalcemia, interacts with the iron-dependent M2 subunit of ribonucleotide reductase (RRM2) and thereby inhibits DNA synthesis.^41^ Interestingly, gallium nitrate has demonstrated antineoplastic activity in various cancers, such as advanced bladder cancer and non-Hodgkin’s lymphoma.^42–44^ So far, experiments with gallium nitrate showed inconsistent results in 2 GBM cell lines.^45^ Here, we demonstrated that high concentrations of up to 2 mM of gallium nitrate result in a heterogeneous and poor response in all GBM cell lines, including the GBM8 primary cell culture.

Lastly, exposure to the XIAP inhibitors AT-406, birinapant, GDC-0152, GDC-0917, and LCL161 showed limited efficacy in the GBM cell lines. This is in contrast to 2 previous preclinical studies. Tchoghandjian et al.^46^ showed that in vitro and in vivo targeting of IAP with GDC-0152 triggered apoptosis in multiple GBM cell lines and improved the outcome in GBM-bearing mice. Furthermore, a comprehensive preclinical study of Zakaria et al.^47^ with birinapant in various GBM cell lines, as a single agent or combined with temozolomide, showed remarkable differences in treatment responses. However, in line with our results, the preclinical study of Houghton et al.^48^ with the Smac mimetic LCL161 demonstrated limited in vitro efficacy, but significant growth delay in a GBM xenograft. These conflicting results in which in vivo responses were better than the in vitro efficacy may demonstrate the critical effect of the tumor microenvironment on therapeutic responses in GBM or could indicate that insufficient target engagement was reached in the tumor.^47,49^

Over the past decades, progress in the improvement of the treatment and survival of patients with GBM has been frustratingly slow due to multiple factors, including the blood–brain barrier, intra- and intertumoral heterogeneity, and the tumor microenvironment. As with most drugs tested in GBM, it is possible that insufficient intracranial concentrations of the antineoplastic agents are reached due to the inadequate delivery across the blood–brain barrier.^50^ Unfavorable physicochemical properties for blood–brain barrier transfer are the relatively large size (molecular weight = 545.64; 8 rotational bonds) as well as the charge (3 hydrogen bond donors) of RGB-286638.^51^ This could explain our conflicting preclinical results with RGB-286638, in which in vitro responses were more promising than the in vivo efficacy. Therefore, RGB-286638 might be subjected to drug discovery to overcome these unfavorable characteristics.

Furthermore, exposure to these drugs may sensitize GBM cells for treatment with temozolomide or radiotherapy. For instance, Wagner et al.^38^ showed that combinational treatment with the Smac mimetic BV6 and temozolomide synergistically reduces cell viability and triggers apoptosis in GBM cells. Similarly, RRM2 inhibition could sensitize cells to temozolomide chemotherapy.^25,26^ Lastly, colleagues have recently demonstrated that the MAPK-targeting agent MEK162 was found to enhance the effect of radiotherapy on GBM cells in their in vitro and in vivo GBM model.^18^ Based on the results of these studies, the efficacy of the antineoplastic drugs targeting RRM2, XIAP, and MAPK9 combined with temozolomide and/or radiotherapy still warrants further investigation.

An important aspect of our work is that we used publicly available gene expression data collected from GEO and TCGA. A significant advantage of this approach is that these databases contain large amounts of data, which have yet to be fully explored in all their details. Therefore, the use and reanalysis of publicly available gene expression data may lead to novel insights and discoveries. Importantly, FGmRNA profiling was applied on mRNA expression data, which should be carefully interpreted, since the FGmRNA expression levels of genes might not always be strongly correlated with the corresponding protein expression levels. For instance, protein levels could be lower due to various cellular processes, such as a high turn-over. Furthermore, it is difficult to distinguish the effect of target gene overexpression in GBM cells from the impact of target gene overexpression in surrounding nontumor cells. To minimalize this effect caused by surrounding nontumor cells, we used sufficient normal brain tissue samples as a reference and created a threshold for overexpression.

In conclusion, with our innovative method of FGmRNA profiling followed by data-driven prioritization, we identified *RRM2*, *MAPK9*, and *XIAP* as potential therapeutic targets for GBM. The MAPK9 inhibitor RGB-286638 showed promising in vitro results and warrants further investigation.

## Supplementary Material

vdaa151_suppl_Supplementary_Figures

vdaa151_suppl_Supplementary_Table_S1

vdaa151_suppl_Supplementary_Table_S2

vdaa151_suppl_Supplementary_Table_S3

vdaa151_suppl_Supplementary_Table_S4

vdaa151_suppl_Supplementary_Methods

## Notes

**Authorship Statement.** All authors were involved in the writing of the manuscript at draft and any revision stages and have read and approved the final version. Conceptualization, C.B., M.L., H.V., A.W., and R.F.; methodology, C.B., U.A., M.H., T.L., B.W., and R.F.; formal analysis, C.B., U.A., M.H., B.W., and R.F.; resources, C.B., U.A., M.H., T.L., B.W., and R.F.; data collection, C.B., U.A., M.H., T.L., B.W., and R.F; writing—original draft preparation, C.B., U.A, M.H., H.V., B.W., A.W., and R.F.; writing—review and editing, C.B., U.A., M.H., M.L., T.L., H.V., B.W., A.W., and R.F.; supervision, H.V., B.W., A.W., and R.F.
