# Supplementary material for: Data-driven prioritization and preclinical evaluation of therapeutic targets in glioblastoma
Source: Neurooncol Adv. 2020 Nov 5;2(1):vdaa151. doi: 10.1093/noajnl/vdaa151 (PMC7764503; doi:10.1093/noajnl/vdaa151)

**SUPPLEMENTARY FIGURES**

**Supp. Fig S1.** Proliferation assay of exposure to **A**) RRM2 inhibitor Gallium nitrate and the XIAP inhibitors **B**) AT-406, **C**) Birinapant, **D**) GDC-0152, **E**) GDC-0917 and **F**) LCL-161 for 72 hours.


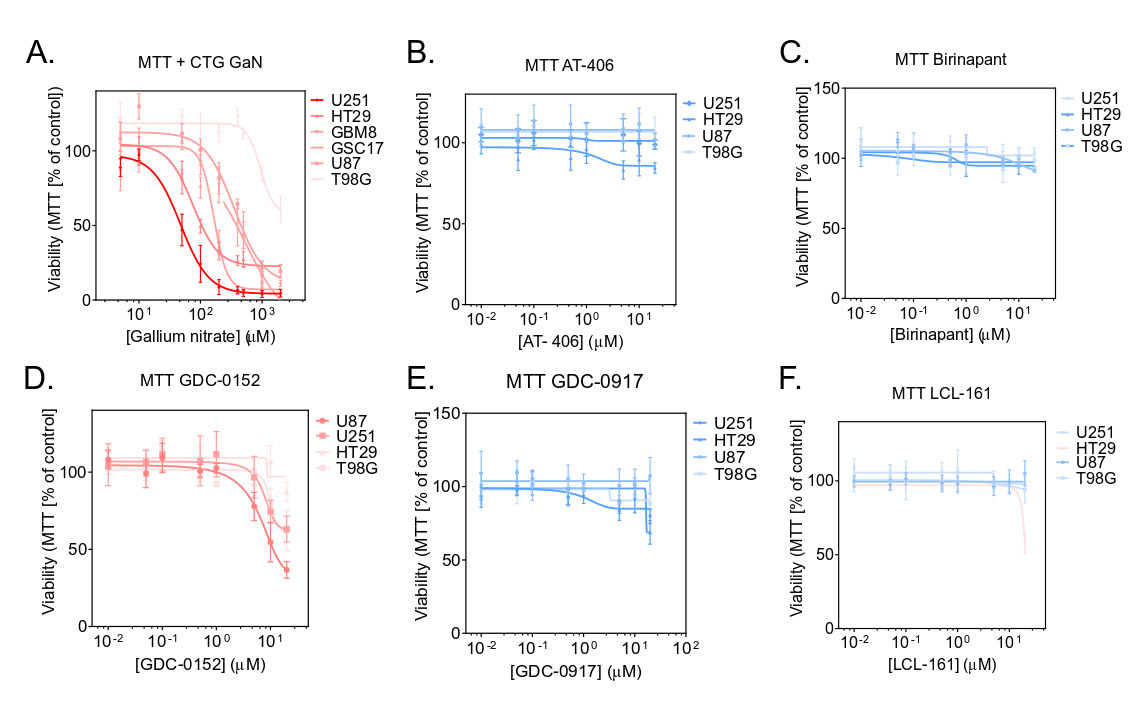


**Supp. Fig S2.** Proliferation assay of exposure to RGB-286638 in all glioblastoma sphere culture model (GSCs)


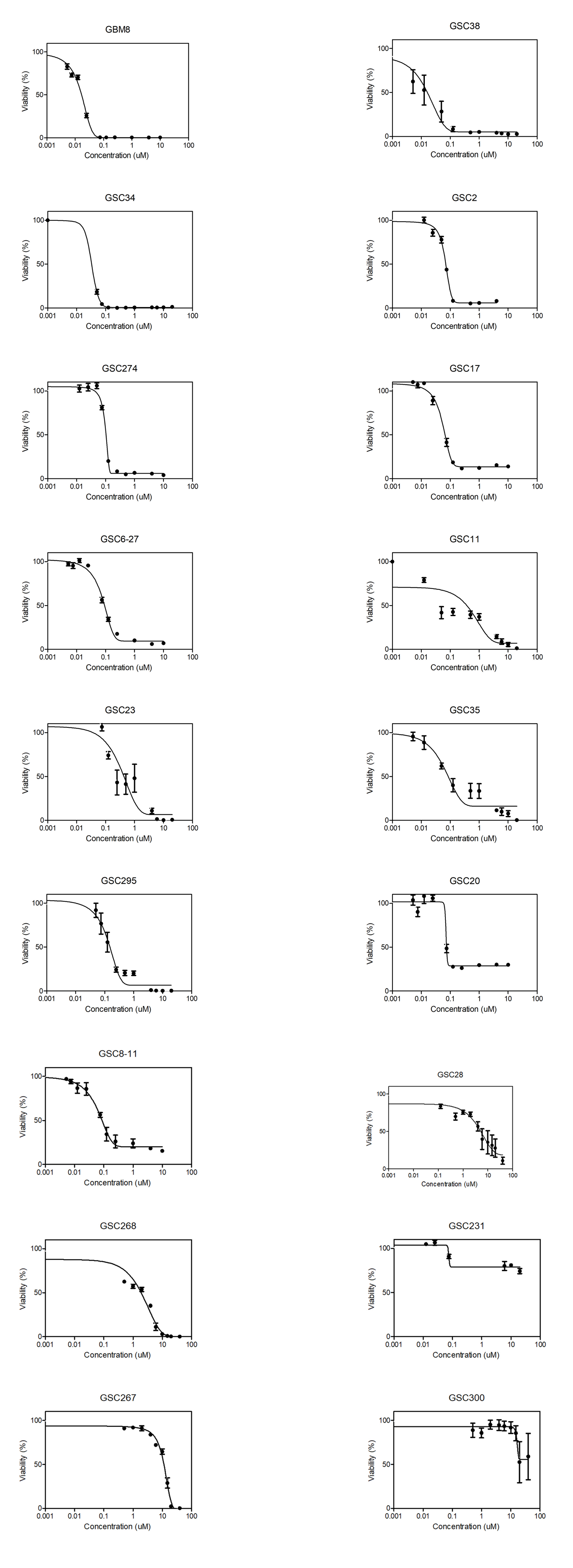

Supplement: vdaa151_suppl_Supplementary_Figures [file vdaa151_suppl_supplementary_figures.docx]
