# Supplementary material for: Data-driven prioritization and preclinical evaluation of therapeutic targets in glioblastoma
Source: Neurooncol Adv. 2020 Nov 5;2(1):vdaa151. doi: 10.1093/noajnl/vdaa151 (PMC7764503; doi:10.1093/noajnl/vdaa151)
Supplement: vdaa151_suppl_Supplementary_Table_S1 [file vdaa151_suppl_supplementary_table_s1.docx]

| **Supplementary table S1.** Keywords used to search SOFT files | |
| --- | --- |
| **Tissue type** | **Keywords** |
| Normal brain tissue | Brain, cerebral |
| Glioblastoma | Glioblastoma, multiforme, GBM, HGG, gliosarcoma, astrocytoma, astroglioma, oligodendroglioma, oligodendroblastoma, glioma, glial |
