# Supplementary material for: Data-driven prioritization and preclinical evaluation of therapeutic targets in glioblastoma
Source: Neurooncol Adv. 2020 Nov 5;2(1):vdaa151. doi: 10.1093/noajnl/vdaa151 (PMC7764503; doi:10.1093/noajnl/vdaa151)
Supplement: vdaa151_suppl_Supplementary_Methods [file vdaa151_suppl_supplementary_methods.docx]

**Data-driven prioritization and preclinical evaluation of therapeutic targets in glioblastoma**

Cyrillo G. Brahm^a,b^, U. Kulsoom Abdul^c,†^, Megan Houweling^c,†^, Myra E. van Linde^b^, Tonny Lagerweij^c^, Henk M.W. Verheul^b,d^, Bart A. Westerman^c^, Annemiek M.E. Walenkamp^a^ and Rudolf S.N. Fehrmann^a^

^a^ Department of Medical Oncology, University of Groningen, University Medical Center Groningen, Groningen, the Netherlands

^b^ Department of Medical Oncology, Cancer Center Amsterdam, Amsterdam University Medical Centers, location VUmc, Amsterdam, the Netherlands

^c^ Department of Neurosurgery, Cancer Center Amsterdam, Amsterdam University Medical Centers, location VUmc, Amsterdam, the Netherlands

^d^ Department of Medical Oncology, Radboud University Medical Center, Nijmegen, the Netherlands

^†^These authors contributed equally to this work.

**Corresponding author:**

Rudolf S.N. Fehrmann, MD, PhD, Department of Medical Oncology, University Medical Center Groningen, Hanzeplein 1, P.O. Box 30.001, 9700 RB Groningen, The Netherlands. Phone: +31 50 361 2620, Fax: +31 50 361 9306, E-mail: r.s.n.fehrmann@umcg.nl

**SUPPLEMENTARY MATERIALS AND METHODS**

*Data acquisition*

We collected publicly available raw microarray expression data from the Gene Expression Omnibus (GEO) [1]. We obtained gene expression data from GEO for samples that were processed on the HG-U133A (GEO accession number GPL96) and HG-U133 plus 2.0 (GEO accession number GPL570) Affymetrix platforms.

*Search strategy*

Simple Omnibus Format Text (SOFT) files were downloaded for the GPL96 and GPL570 platforms. These SOFT files are simple line-based formats with plain text and contain information on the samples as provided by the investigator who uploaded the data to GEO. Only glioblastoma-related samples were considered relevant for this study. Therefore, to collect samples from GEO that could possibly be related to GBM, we filtered the samples based on the content of their description field. We used the following keywords to filter the samples: *‘glioblastoma’, ‘multiforme’, ‘GBM’, ‘HGG’, ‘gliosarcoma’, ‘astrocytoma’, ‘astroglioma’, ‘oligodendroglioma’, ‘oligodendroblastoma’, ‘glioma’, ‘glial’, ‘brain’, ‘cerebral’*. Our search strategy was aimed at sensitivity to minimize the chance of missing relevant samples. Therefore, all samples had to be manually checked to remove all non-relevant samples. Only samples of fresh frozen normal or malignant brain tissue were included for further analysis. Cell lines, cultured samples, and post-mortem or animal tissues were excluded.

*Data acquisition The Cancer Genome Atlas (TCGA)*

In addition, we collected raw gene expression profiles from the TCGA Glioblastoma multiforme dataset. These profiles were generated with the Affymetrix HT HG-U133A. This platform contains identical probes as the Affymetrix HG-U133A obtain from GEO. This enabled us to integrate the TCGA dataset with the GEO dataset.

*Sample processing*

After automatic filtering and manual curation, all non-corrupted, available CEL files were downloaded. Since numerous samples have been uploaded to GEO multiple times, we created a MD5 hash string for every CEL file. A MD5 hash string is a 32-digit hexadecimal number, which is generated from the binary content of a file. This MD5 hash string, like an unique fingerprint for all the CEL files, was used to identify duplicate samples. After removal of all the duplicates, pre-processing and normalization was performed according to the robust multi-array average (RMA) algorithm with RMAExpress (version 1.1.0) using the latest available Affymetrix GeneChip Array CDF layout files [2].

*Sample Quality Control*

A principle component analysis (PCA) was used on the sample correlation matrix as quality control. The first principal component (PC_qc_) accounts for as much of the variability in the data as possible. The amount of variance explained by the PC_qc_ is roughly 80 – 90%. This variance is the result of a platform-specific effect, rather than a probe-specific effect. We assumed that samples of poor quality would have a lower correlation with the PC_qc_ and, therefore, excluded all the samples with a correlation *R* < 0.8 [3].

*Functional genomic mRNA profiling*

For a detailed description of functional genomic mRNA (FGmRNA) profiling we refer to Fehrmann *et al* [4]. In short, we performed a PCA on expression profiles of 77.840 publicly available samples and identified a large set of robust principle components (PCs), defined as transcriptional components (TCs). We found that a limited number of TCs capture a large proportion of the variance seen within the mRNA transcriptome. Subsequently, we identified a subset of these TCs that were associated with non-genetic regulatory factors. On correcting expression profiles for these non-genetic TCs, we observed that the residual expression levels (*i.e.* FGmRNA-profile) correlated strongly with copy number alterations occurring on the DNA level. Thus, FGmRNA-profiling gives us an enhanced view of the downstream consequences of genomic alterations occurring in tumor genomes on gene expression levels.

*Removing batch effects*

We performed an PCA on the sample correlation matrix calculated with the generated FGmRNA-profiles. We observed that the first three PCs clearly captured differences in FGmRNA expressing signals that could be attributed to the three different platforms present in the data set. Subsequently, we corrected the FGmRNA-profiles by removing the variance captured by these first three PCs.

*Class comparison*

We used the FGmRNA-profiles of healthy brain tissue and glioblastoma tissue to perform a genome-wide class comparison analysis. A Welch’s T-test was used to identify genes with differential FGmRNA-expression. To assess the degree of multiple testing, we performed this analysis within a multivariate permutation (MVP) test (1.000 permutations) with a false discovery rate of 1% and a confidence level of 99% [5]. This results in a list of significantly associated genes, which contains no more than 1% false positives.

*Literature search*

To compare the list of upregulated genes identified with the class comparison between FGmRNA-profiles of glioblastoma and healthy brain tissue with known immunohistochemistry (IHC) and mRNA expression data from literature, PubMed was searched for articles published in English until March 2016. The corresponding HUGO gene symbols of the upregulated genes were used in our search strategy, together with the following search terms: ‘*glioblastoma*’, ‘*GBM*’, ‘*brain*’, ‘*tumor*’, ‘*cancer*’, ‘*neoplasm*’ and ‘*oncogenic*’ in various combinations.

*Drug-Gene Interaction Database*

To determine which genes might be interesting as a therapeutic target for GBM treatment, we explored the identified set of upregulated genes with the use of the Drug-Gene Interaction Database (DGIDb; dgidb.genome.wustl.edu). The DGIDb integrates data of disease-relevant human genes, drugs, drug-gene interactions, and potential druggability from 13 primary sources [6]. The DGIDb classifies genes of the druggable genome into two main categories. The first group involves genes with known drug interactions based on literature and publicly available databases. The second group includes genes that currently may not be targeted therapeutically, but are potentially druggable based on their relation and position in certain gene categories associated with druggability (*e.g.* kinases). Based on the identified upregulated genes for which a drug-gene interaction was found, we performed an additional literature search to determine the potential therapeutic applicability of these genes. The identified upregulated genes were considered as potentially therapeutic applicable if scientific evidence with registered or experimental drugs targeting these genes was available (*e.g.* *ex vivo or in vivo* experiments and clinical trials). Therefore, PubMed was used to search for articles published in English and clinicaltrials.gov was used to review all finished and ongoing clinical trials. The HUGO gene symbol or generic drug name were used in various combinations with the following search terms: ‘*glioblastoma*’, ‘*GBM*’, ‘*brain*’, ‘*tumor*’, ‘*cancer*’, ‘treatment’, ‘therapy’. Subsequently, we divided the identified genes in the following categories: 1) Anti-neoplastic drug targets in clinical newly diagnosed or recurrent GBM trials, 2) Anti-neoplastic drug targets in clinical trials with solid tumors, 3) Drug targets in preclinical cancer models.

**REFERENCES**

[1] Barrett T, Wilhite SE, Ledoux P, et al. NCBI GEO: archive for functional genomics data sets--update. *Nucleic Acids Res* 2013 Jan,**41**(Database issue), D991-D995.

[2] Irizarry RA, Hobbs B, Collin F, et al. Exploration, normalization, and summaries of high density oligonucleotide array probe level data. *Biostatistics* 2003 Apr,**4**(2), 249-264.

[3] Smith LI. A tutorial on Principal Components Analysis. 2002.
Available at: <http://classifion.sicyon.com/References/princomp.pdf>.

[4] Fehrmann RS, Karjalainen JM, Krajewska M, et al. Gene expression analysis identifies global gene dosage sensitivity in cancer. *Nat Genet* 2015 Feb,**47**(2), 115-125.

[5] Chung EY, Romano JP. Multivariate and Multiple Permutation Tests. 2013.
Available at: <http://statistics.stanford.edu/sites/default/files/2013-05_0.pdf>.

[6] Griffith M, Griffith OL, Coffman AC, et al. DGIdb: mining the druggable genome. *Nat Methods* 2013 Dec,**10**(12), 1209-1210.
